# Supplementary material for: Identification and functional analysis of non-coding regulatory small RNA FenSr3 in Bacillus amyloliquefaciens LPB-18
Source: PeerJ. 2023 May 15;11:e15236. doi: 10.7717/peerj.15236 (PMC10194069; doi:10.7717/peerj.15236)
Supplement: Supplemental Information 4 [file peerj-11-15236-s004.zip › KO/CK-vs-T1_map/map05033.html]

KEGG PATHWAY: Nicotine addiction - Reference pathway


|  |  |
| --- | --- |
| **Nicotine addiction - Reference pathway** |  |

[
Pathway menu
| Organism menu
| Pathway entry
| Show description
| User data mapping
]

Nicotine is one of the main psychoactive ingredients in tobacco that contributes to the harmful tobacco smoking habit. A common feature of addictive drugs, including nicotine, is that they increase dopamine (DA) release in the nucleus accumbens (NAc). The principal dopaminergic projections to NAc arise from neurons in the ventral tegmental area (VTA). In the VTA, alpha6- and alpha4beta2-containing nAChRs (alpha6\*-AChRs and alpha4beta2\*-AChRs, respectively) are located on GABAergic terminals and provide inhibitory inputs onto DAergic neuons, while alpha7\*-nAChRs are located on glutamatergic terminals and activation of these receptors enhances glutamate release and increases excitability of DAergic neurons. Nicotine acts as an agonist to activate and desensitize these nicotinic acetylcholine receptors (nAChRs). After a short exposure to nicotine, alpha6\*- and alpha4beta2\*- nAChRs on GABAergic afferents are desensitized, decreasing GABA release and decreasing local inhibition of DA neurons. But the alpha7\*-nAChRs on glutamatergic afferents remain active and enhance glutamate excitation of the DA neurons, leading to increased DA release in the NAc -and so facilitate the reinforcing effects of nicotine.

|  |  |  |
| --- | --- | --- |
| Reference pathway | 184% 150% 122% 100% 82% 67% 55% | 图片下载 |
